# Cognitive and behavioral disturbances in PSP and MSA: clinical features and imaging correlates

**DOI:** 10.1007/s00702-026-03146-8

**Published:** 2026-05-11

**Authors:** I. Ruiz-Barrio, A. Horta-Barba, S. Martinez-Horta, J. Kulisevsky, J. Pagonabarraga

**Affiliations:** 1 Facultad de Medicina, Universitat Autònoma de Barcelona (UAB), Barcelona, Spain; 2 Movement Disorders Unit, Neurology Department, Hospital de La Santa Creu I Sant Pau, Mas Casanovas 90, 08041 Barcelona, Spain; 3 Sant Pau Biomedical Research Institute (IIB-Sant Pau), Barcelona, Spain; 4 Centro de Investigación en Red-Enfermedades Neurodegenerativas (CIBERNED), Madrid, Spain

**Keywords:** Progressive supranuclear palsy, Multiple system atrophy, Neuroimaging, Cognition, Behavior

## Abstract

Cognitive and behavioral disturbances are increasingly recognized as core features of Progressive Supranuclear Palsy (PSP) and Multiple System Atrophy (MSA), with important clinical and prognostic implications. In PSP, cognitive impairment—often an early sign—is dominated by a dysexecutive syndrome linked to dorsolateral prefrontal atrophy and fronto-subcortical circuit disruption. Deficits also extend to attention, memory, language, visuospatial abilities, and social cognition. Apathy is the most common behavioral symptom, associated with medial prefrontal and subcortical atrophy, while impulsivity, disinhibition, and mood symptoms reflect broader orbitofrontal and posterior cortical involvement. Imaging studies highlight frontooccipital white matter disruption and subcortical tau deposition as key contributors to these features. In MSA, cognitive impairment is typically milder and more variable, most commonly involving executive dysfunction related to fronto-subcortical and cerebellar degeneration. Memory and visuospatial issues usually stem from impaired executive control. Behavioral symptoms—especially apathy and depression—are frequent but underrecognized. Frontal and cerebellar atrophy, along with disrupted striato-frontal connectivity and reduced metabolism, correlate with these changes. Autonomic dysfunction, particularly orthostatic hypotension, may further exacerbate cognitive decline through cerebral hypoperfusion. These findings underscore the importance of early detection of cognitive and behavioral symptoms in PSP and MSA. Given their significant impact on quality of life and functional decline, comprehensive assessment tools and multidisciplinary care approaches are essential to improve outcomes.

## Introduction

Progressive supranuclear palsy (PSP) is a neurodegenerative disease that, from a neuropathological standpoint, represents a primary four-repeat tauopathy (Kovacs et al. 2020). Clinically, it is classified both as an atypical Parkinsonian syndrome (APS) and as part of the frontotemporal dementia (FTD) spectrum, as it shares motor, cognitive, and behavioral characteristics of both groups (Sha et al. 2006; Murley et al. 2020; Bruno et al. 2024). The classic phenotype of PSP, originally described as Richardson's syndrome (RS), is characterized by early postural instability with recurrent falls, axial rigidity, bradykinesia, dysarthria, and vertical supranuclear gaze palsy. Although cognitive and behavioral disturbances were noticed in the earliest clinical descriptions, they were not initially regarded as core diagnostic features of PSP (Hoglinger et al. 2017). Over time, however, cognitive symptoms and behavioral changes have gained increasing recognition, and they are now acknowledged as highly prevalent, often present from the early disease stages, and to play a significant role in functional disability (Hoglinger et al. 2017). The description of individuals with PSP presenting with initial symptoms within the FTD spectrum led to the definition of new PSP subtypes in the 2017 PSP diagnostic criteria (Hoglinger et al. 2017). In addition to PSP-RS, these criteria provided formal diagnostic definitions for previously described cortical variants, such as PSP with predominant speech/language impairment (PSP-SL), frontal presentation (PSP-F), and PSP with corticobasal syndrome features (PSP-CBS), among others. This expansion has increased the sensitivity of PSP diagnosis by encompassing a broader range of clinical phenotypes in which cognitive and behavioral symptoms are primary features (Hoglinger et al. 2017; Jabbari et al. 2020). Moreover, clinical studies have shown that PSP subtypes evolve dynamically over the course of the disease, observing that cognitive decline and the development of behavioral changes occur across all PSP variants as the condition progresses.

Multiple system atrophy (MSA) is a neurodegenerative disorder that neuropathologically belongs to the group of synucleinopathies, with predominant accumulation of α-synuclein in oligodendroglial cells (Krismer and Wenning 2017). Clinically, MSA encompasses a spectrum of phenotypes, including Parkinsonian (MSA-P) and cerebellar (MSA-C) variants, typically accompanied by early and prominent autonomic dysfunction, which together constitute the core diagnostic features of the disease (Wenning et al. 2022). Historically, MSA has been regarded as a movement disorder with minimal cognitive involvement, and dementia has traditionally been considered an exclusion criterion for diagnosis. However, accumulating evidence over the past decade has challenged this view, demonstrating that mild cognitive impairment (MCI) is relatively common in MSA and may be present even in the early stages of the disease (Stankovic et al. 2014). Although cognitive decline in MSA is generally less prevalent, less severe, and progresses more slowly than in PSP, both conditions may share certain overlapping features (Fiorenzato et al. 2019).

## Cognition and behavior in PSP

Cognitive changes occur in 50–90% of patients with PSP, often within the first year of the disease (Horta-Barba et al. 2021). The first cognitive description in PSP was based on PSP-RS subtype. It was characterized by impairment in attention, executive functions, and processing speed. But now, the PSP cognitive profile has expanded to a more widespread impairment including language dysfunction, involvement of visuospatial skills, and social cognition, among other domains (Horta-Barba et al. 2021). Executive function is considered the most frequently and early affected domain, with no significant differences between clinical subtypes. Around 80% of PSP patients show pathological scores in Frontal Assessment Battery (FAB) (Horta-Barba et al. 2021). Notably, these estimates derive from early PSP cohorts, indicating that executive dysfunction is detectable at stages when diagnostic uncertainty with other APS is still common. This patients usually have dramatically slowed information processing speed, with problems in orienting attentional resources, difficulty in planning, and shifting conceptual sets, and prominent recall deficits with moderate forgetfulness (Horta-Barba et al. 2021; Litvan et al. 1989; Grafman et al. 1995; Street et al. 2022). In addition to these attentional and executive deficits, more than 50% of PSP patients will show language dysfunction that may include impaired verbal fluency, word-finding difficulty, and, in some variants, more severe speech and language deficits (Catricalà et al. 2019). Although fundamental comprehension is often preserved initially, approximately three quarters of patients exhibit articulatory or motor speech abnormalities, while semantic fluency is impaired in nearly 60% of cases. These linguistic difficulties may be especially prominent in subtypes like PSP-SL, where speech and language capacity are selectively affected (Catricalà et al. 2019; Busteed et al. 2024; Peterson et al. 2021). So, speech–language impairment may constitute the initial clinical presentation in PSP-SL but can also appear later in the disease course of other PSP subtypes. Visuospatial impairment, including altered depth perception and orientation, are likewise a frequent discovery, with up to 40–70% of patients showing deficits on standard visuospatial tests. This feature, in combination with supranuclear gaze palsy, may contribute substantially to fall risk (Horta-Barba et al. 2021; Smith et al. 2021). Finally, social cognition is frequently impaired in progressive supranuclear palsy. Clinically relevant social difficulties are reported by approximately half of patients, and formal assessment reveals deficits in emotion processing and mentalizing, including increased errors in facial emotion recognition and marked impairment on theory-of-mind tasks such as *faux pas* detection or sarcasm comprehension (Burrell et al. 2014; De Souza et al. 2022; Ghosh et al. 2012).

Alongside cognitive symptoms, PSP also presents with neuropsychiatric disturbances such as apathy and impulsivity, which contribute to disability throughout the disease course. Apathy is seen in approximately 65–90% of PSP patients and can be an early manifestation of the disease (Horta-Barba et al. 2021; Cuoco et al. 2023). Characterized by diminished motivation, reduced goal-directed activity, and obvious blunting of emotional reactivity, apathy can significantly impair activities of daily living (Pagonabarraga et al. 2015; Cuoco et al. 2022). Regarding impulsivity, patients often exhibit restlessness, changes in eating behaviors such as hyperorality or altered food preferences, vocal and motor stereotypies, including repetitive or purposeless movements. The severity of these symptoms varies between PSP phenotypes, although it becomes nearly universal as disease evolves. Apathy and impulsivity are more pronounced and develop earlier in cortical variants compared to subcortical ones, such as PSP-P (Ruiz-Barrio et al. 2024), and it is very frequent and severe in PSP-RS. Additionally, deficits in empathy are frequently observed, contributing to the disruption of interpersonal relationships and social functioning (Horta-Barba et al. 2021). Disinhibition, though less frequent than apathy, is still a prominent PSP behavioral symptom. Patients may display impulsive judgment, socially inappropriate comments, or activities that depart from the former personality standards. These manifestations are more pronounced in frontal variants (PSP-F) (Ruiz-Barrio et al. 2024). Among behavioral changes, irritability is also a common manifestation, usually expressed as frustration with mundane activity or interpersonal interaction. Depression is also common, with reported prevalence rates of up to 75%, particularly in patients who have adequate insight to appreciate their reduced functionality, resulting in enhanced negative affect and social withdrawal (Cuoco et al. 2023). Although psychotic symptoms such as hallucinations and delusions are not a prominent aspect of PSP's profile, they do occur in approximately 10% of cases, either in advanced stages or in association with concomitant medication (e.g., dopaminergic treatments) (Donker Kaat et al. 2007). Emotional lability—sudden and uncontrollable crying or laughter—has been documented in PSP and may also occur in MSA. In the absence of other frontal features, it may be more suggestive of MSA. (Horta-Barba et al. 2021).

Although no single instrument fully captures the entire cognitive–behavioral spectrum of PSP, a disease-specific bedside tool has recently been developed, namely the Short Cognitive and Neuropsychiatric (ShoCo) scale. This brief composite instrument has demonstrated good convergent validity with established cognitive measures, sensitivity to longitudinal change and feasibility for routine clinical use (Porsche et al. 2025). Together with bedside clinical assessment of frontal signs associated with cognitive dysfunction in PSP (Ruiz-Barrio et al. 2024), these approaches facilitate a more PSP-specific evaluation of non-motor features relevant for diagnosis, functional prognosis, and disease monitoring (Table 1).

**Table 1:** Clinical cognitive and behavioural features in PSP and MSA

Domain	PSP	MSA	Differential diagnostic value	Neural correlates	References
*Cognitive*					
Overall cognitive impairment	Cognitive changes in 50–90%, often within the first year	MCI in 44–60%; dementia 5–26%; cognitive decline less prevalent, less severe and slower than PSP	Earlier and more prominent cognitive syndrome supports PSP	Predominant involvement of frontal and frontal–subcortical networks in PSP; heterogeneous and incompletely understood substrates in MSA, with possible contributions from frontal, limbic, subcortical, and cerebellar structures	Kovacs et al. 2020; Fiorenzato et al. 2019; Horta-Barba et al. 2021; Jellinger 2023; Li et al. 2021a; Jellinger 2024; Ali et al. 2024; Kvickström et al. 2011; Agosta et al. 2014; Jellinger 2020)
Executive dysfunction	Most frequent, early and severely impaired domain; around 80% pathological FAB; detectable in early PSP cohorts	Most prevalent cognitive feature, usually mild, in more than 40%	An early and prominent dysexecutive syndrome is more suggestive of PSP	In PSP, associated with degeneration of medial and lateral prefrontal cortex and frontal–subcortical circuits. In MSA, linked to frontal cortical atrophy, subcortical structures, and cerebellar involvement	Horta-Barba et al. 2021; Asi et al. 2014; Wenning and Brown 2009; Sambati et al. 2020; Zhang et al. 2018; Ali et al. 2024; Kvickström et al. 2011; Agosta et al. 2014; Fiorenzato et al. 2017; Kim et al. 2015; Dash et al. 2022)
Attention/processing speed	Very slowed information processing and attentional orientation deficits	Attention/processing speed commonly impaired, usually mild	A severe attention and processing speed impairment is more suggestive of PSP	Dysfunction of frontal–subcortical circuits, including prefrontal cortex and connected subcortical structures; in MSA, possible contribution of thalamic and cerebellar pathways	Horta-Barba et al. 2021; Street et al. 2022; Lazzeri et al. xxxx), (Pang et al. 2024)
Memory impairment	Not a typical feature of PSP. Most often retrieval deficits secondary to executive and attentional dysfunction rather than a primary amnestic syndrome	Not a typical feature of MSA. Reported in a minority of patients (11–16%) and generally mild; Usually reflect disrupted encoding and retrieval strategies related to executive dysfunction	Not a strong discriminator	No consistent evidence of primary hippocampal pathology	Horta-Barba et al. 2021; Litvan et al. 1989; Grafman et al. 1995; Street et al. 2022; Zhang et al. 2018; Jellinger 2025; Šubert et al. 2025; Kawai et al. 2008)
Language deficits	Common, > 50% language deficits, more prominent as the disease progress. In PSP-SL may be initial presentation	Core language functions generally relatively preserved, communication difficulties more often related to motor speech impairment	Language impairment favors PSP	In PSP-SL, predominant involvement of prefrontal and language-related cortical regions with relatively less midbrain atrophy compared with typical PSP; limited data on specific neural substrates in MSA	Stankovic et al. 2014; Grafman et al. 1995; Street et al. 2022; Catricalà et al. 2019; Cuoco et al. 2022; Sambati et al. 2020; Klos et al. 2005)
Visuospatial impairment	Frequent; 40–70% abnormal on standard visuospatial tests	Not frequent, around 15–30% and milder than PSP	Prominent visuospatial dysfunction favors PSP	In PSP, disruption of distributed cortical networks, including fronto-parietal pathways; limited data on specific neural substrates in MSA	Stankovic et al. 2014; Koga et al. 2017a; Asi et al. 2014; Sambati et al. 2020; Zhang et al. 2018)
*Behavioral*					
Social cognition	Clinically relevant social difficulties in around 50%; deficits in emotional recognition and theory of mind	Not a commonly reported feature	Social cognition deficits favor PSP	In PSP, altered frontal and limbic networks involved in emotion processing and theory of mind	Peterson et al. 2021; Smith et al. 2021; Burrell et al. 2014)
Apathy	Very frequent, around 65–90%, usually an early feature	Frequent but less prominent than in PSP	Severe and early apathy supports PSP	Strongly associated with medial frontal and orbitofrontal cortex degeneration and their subcortical connections, including striatal structures; similar mechanisms suggested but less well characterized in MSA	Stankovic et al. 2014; De Souza et al. 2022; Ghosh et al. 2012; Cuoco et al. 2023; Jellinger 2025; Kawai et al. 2008; Jellinger 2024; Kvickström et al. 2011; Cordato et al. 2006; Aarsland et al. 2001; Josephs et al. 2011)
Depression	Up to 75%, especially when insight preserved	Present in 30–70% behavioural symptom cluster; fluctuating course in MSA-P	Not a strong discriminator	In PSP, associated with widespread cortical thinning, particularly in posterior associative regions; in MSA, affective symptoms linked to fronto-cingulate and limbic structures	De Souza et al. 2022; Jellinger 2025; Kawai et al. 2008; Jellinger 2024; Kvickström et al. 2011)
Irritability/emotional lability	May be part of the behavioural spectrum of PSP	Emotional incontinence is considered a supportive non-motor feature according to current diagnostic criteria. It may be more prominent in the cerebellar subtype (MSA-C)	It may occur in both PSP and MSA, but in absence of other frontal features, more suggestive of MSA	Likely related to dysfunction of frontal–limbic circuits; in MSA, possible involvement of cerebello–limbic pathways, particularly in MSA-C	Jellinger 2025; Kawai et al. 2008; Jellinger 2024)

## Cognition and behavior in MSA

The prevalence of mild cognitive impairment (MCI) in MSA is estimated to be 44–60% (Jellinger 2023; Li et al. 2021a). However, dementia is less prevalent than in PSP and does not exhibit a similarly rapid rate of progression (Fiorenzato et al. 2019; Koga et al. 2017a), with autopsy-based series indicating dementia in 5–26% of MSA cases. Similarly to PSP, frontal–executive dysfunction represents the most prevalent cognitive feature in MSA, affecting over 40% of patients. This include planning deficits, problem-solving deficits, and deficits in complex cognitive flexibility, that may be severe enough to cause impairment of daily functioning (Asi et al. 2014; Wenning and Brown 2009; Sambati et al. 2020; Zhang et al. 2018). In PSP, attention–dysexecutive symptoms may represent among the earliest presenting manifestations and can begin years before diagnosis, while in MSA cognitive impairment may occur early but is more often reported as becoming prominent with longer disease duration. Processing speed of information and attention are also commonly impaired, with patients experiencing difficulty with sustained attention and with slowing of overall mental processes. Although memory impairment in MSA is generally less pronounced than in Alzheimer's disease, with reported rates of 11–16%, executive dysfunction may disrupt encoding and retrieval strategies so as to produce working memory and free recall impairment (Zhang et al. 2018; Jellinger 2025; Šubert et al. 2025; Kawai et al. 2008). While language abilities often remain intact early on, around 60–80% of patients may experience dysarthria or forced speech that can progress to partial or even total mutism. In such advanced stages, assistive communication devices and alternative communication strategies often become crucial to maintaining meaningful interaction. However, in contrast to PSP, core language functions are generally relatively preserved in MSA, particularly in early stages, and communication difficulties more commonly reflect motor speech impairment rather than primary linguistic deficits. Accordingly, the presence of early and prominent language impairment may be helpful in the differential diagnosis between PSP and MSA, favoring PSP when linguistic deficits extend beyond motor speech dysfunction (Horta-Barba et al. 2021; Jellinger 2024). Visuospatial deficits, though typically milder than those seen in PSP, can add to this cognitive burden with a prevalence of 15–30% by impairing orientation and depth perception (Zhang et al. 2018; Jellinger 2025; Šubert et al. 2025; Kawai et al. 2008). Given that visuospatial deficits are less frequently reported in MSA and are generally milder than in PSP, prominent early visuospatial impairment may favor PSP over MSA in the differential diagnosis, particularly when accompanied by early oculomotor signs. When cognitive profiles are compared across MSA subtypes, several studies indicate that patients with the MSA-P variant tend to exhibit more pronounced cognitive impairment, particularly affecting executive, attentional, visuospatial, and language domains (Li et al. 2021a). However, other studies have not observed such differences between MSA-P and MSA-C patients (Kawai et al. 2008; Koga et al. 2017b).

Among the behavioral symptoms of MSA, anxiety, apathy, depression and inflexibility are particularly notable. These symptoms are reported in 30–70% of patients and are often associated with disease duration and severity (Jellinger 2024; Zhang et al. 2019). However, longitudinal studies found that the progression of these manifestations may occur independently of the progression of motor, autonomic, and cognitive symptoms in patients with MSA-P. Some symptoms, such as depression, have been reported as more frequent in MSA-P compared to MSA-C. Additionally, in MSA-P patients, while anxiety and depression may follow a fluctuating course throughout the disease, apathy tends to show a linear progression over time (Jecmenica-Lukic et al. 2021). Finally, addictive and compulsive behaviors related to impulse control disorder and dopamine dysregulation syndrome have also been reported in association with dopaminergic replacement therapy in some MSA patients (Klos et al. 2005; Cilia et al. 2014).

## Neural correlates of cognitive and behavioral symptoms in PSP

Advances in neuroimaging techniques have provided critical insights into the structural and functional brain alterations underlying cognitive dysfunction, apathy, depression, impulsivity, and disinhibition in PSP. Cognitive dysfunction in PSP primarily stems from impairments in both attentional and executive functions that are dependent on the lateral and medial regions of the prefrontal cortex. Sustained attention, planning, and working memory are affected as much as cognitive processes involved in set-shifting, go-no go tasks, and the monitoring, inhibition, and reversal of automatic responses to novel environmental stimuli. Consistent with these findings, volumetric MRI studies have demonstrated that patients with PSP exhibit greater gray matter volume loss in both the medial and lateral prefrontal cortices compared to those with MSA and PD. This atrophy correlates with the severity of executive dysfunction but not with motor impairments. Furthermore, in PSP subgroups characterized by predominant cognitive symptoms versus Parkinsonian features, a significantly higher degree of bilateral prefrontal atrophy has been observed (Ali et al. 2024). These findings support the hypothesis that cognitive impairment in PSP is directly linked to degeneration of the prefrontal cortex, alongside the involvement of subcortical nuclei. PSP with prominent language impairment (PSP-SL) have less prominent midbrain atrophy, but more marked prefrontal atrophy as compared with typical PSP (Peterson et al. 2021; Rohrer et al. 2010).

Using diffusion tensor tractography, selective degeneration of the inferior fronto-occipital fasciculus was found to be associated with severe frontal cognitive symptoms, as well as with apathy and personality changes (Kvickström et al. 2011). Subsequent studies also observed that involvement of the right superior longitudinal and inferior longitudinal fasciculus, left uncinate, and corpus callosum were the best predictors of executive dysfunction in PSP (Agosta et al. 2014). Behavioral changes are also a frequent and characteristic feature in PSP (Cordato et al. 2006), closely related to frontal network dysfunction. Almost all patients suffer from moderate to severe apathy and many of them may exhibit associated disinhibition. Depression, anxiety, and irritability are also common and have a major impact on quality of life (Aarsland et al. 2001). Initial studies showed that various regions of the frontal lobe contributed to behavioral changes, but the lateral posterior frontal cortex particularly correlated with the total Frontal Behavioral Inventory score, and the presence of apathy correlated with atrophy of the putamen (Josephs et al. 2011).

Apathy is significantly associated with executive dysfunction, suggesting that both cognitive dysfunction and apathy in PSP are mediated by degeneration in similar prefrontal areas or by dysfunction of similar frontal-subcortical connections (Cordato et al. 2002). PSP is characterized by more severe apathy and less depression than PD or Alzheimer’s disease. Consistent relationship of apathy with disinhibition gives more evidence about the important role of the dysfunction of the orbitofrontal and medial frontal circuits in the behavioral disturbances of PSP, which has been replicated in subsequent neuroimaging studies showing frontal atrophy studies showing frontal atrophy in volumetric MRI studies to correlate with behavioral changes in PSP (Lansdall et al. 2017). Apathy is associated with atrophy of the ventromedial orbitofrontal cortex, atrophy of the anterior cingulate and ventrolateral orbitofrontal cortex, whilst emotional blunting is more specifically associated with atrophy of the left accumbens and insula. These findings further support the involvement of the medial frontal regions and insular cortex in apathy and suggest that the behavioral and emotional dimensions of the apathy syndrome may arise from distinct neuroanatomical substrates (Stanton et al. 2013). In PSP, greater atrophy in the anterior cingulate cortex, posterior inferior frontal gyrus, amygdala and hippocampus has been also associated with emotional dysregulation, including anxiety, irritability, and agitation/aggression (Cuoco et al. 2023).

Only one study has investigated the neural correlates of depression in PSP. Interestingly, as an emotional complication different from apathy, depression was linked to widespread thinning of the cortex, especially in posterior associative areas. Significant reductions in cortical thickness were found in the right temporo-occipital cortex, left parieto-temporal regions, posterior cingulate cortex, and the precuneus (Urso et al. 2022).

Behavioral changes, related mostly to disinhibited and impulsive behaviors, and impairment in social judgment and behaviors, have been associated with widespread changes in both grey and white matter of the temporal pole, frontal pole, dorsal-anterior cingulate cortex, orbitofrontal and medial frontal cortex and their connecting tracts (Santillo et al. 2016; Lansdall et al. 2018). The assessment of various signs of frontal dysfunction and frontal release (i.e. grasping, groping, anosognosia) showed a correlation with frontal cognitive dysfunction and behavioral changes in PSP patients, appeared as predictors of early functional disability and mortality, and their presence was associated with significant thinning of bilateral middle frontal cortex, right superior frontal cortex, right middle temporal gyrus and right precuneus (Ruiz-Barrio et al. 2024). Finally, it is interesting to describe the findings of studies analyzing the cortical functional changes associated with pathological changes in PSP. Higher amounts of tau in the globus pallidus internus and dentate nucleus have been associated with reduced blood flow in the limbic cortex and prefrontal areas (Theis et al. 2024). Using ^18^F-PI-2620 tau-PET, higher subcortical tau deposition was associated with overall lower cortical perfusion, particularly in the lateral prefrontal and anterior cingulate cortex. These studies suggest that subcortical tau pathology may induce cortical dysfunction, which may contribute to clinical disease manifestation and clinical heterogeneity (Roemer et al. 2024).

## Neural correlates of cognitive and behavioral symptoms in MSA

### Neuropathological considerations

From a neuropathological perspective, there is insufficient evidence to elucidate the neuropathological basis of cognitive decline in MSA. Some studies have identified an association between involvement of limbic structures such as the dentate gyrus and cognitive impairment in these patients (Koga et al. 2017a). Others have found that the combination of Alzheimer's co-pathology along with Lewy body co-pathology may play a role in the cognitive decline of some cases, particularly in the elderly. In contrast, isolated Alzheimer's or Lewy body co-pathology alone does not seem to have such a significant role (Jellinger 2024; Jellinger 2020). Although advanced stages of Alzheimer's pathology are indeed associated with dementia in patients with MSA, this association remains an infrequent finding. Nonetheless, some studies have found that carrying the APOEε4 allele may constitute a risk factor for cognitive decline in MSA (Nasri et al. 2022).

### Neuroimaging correlates

Structural MRI studies have demonstrated greater frontal atrophy in MSA patients with cognitive impairment, particularly in the dorsolateral prefrontal cortex (Fiorenzato et al. 2017). The MSA-P subtype with cognitive decline has also been associated with cortical atrophy in the paracentral and parahippocampal regions (Kim et al. 2015). At the subcortical level, the insula, thalamus, and caudate seem to play a role in executive and attentional dysfunction in MSA patients (Lazzeri et al. xxxx). Finally, a correlation has also been found between cerebellar atrophy and cognitive dysfunction in both MSA-P and MSA-C cases. Specifically, cerebellar atrophy has been linked to impairments in executive dysfunction, verbal memory, new learning, and overall memory (Dash et al. 2022). Regarding behavioral manifestations, studies have found an association between affective symptoms and atrophy of cortical structures such as the fronto-cingulate cortex and left parietal cortex, as well as subcortical structures such as the amygdala and ventral striatum (Jellinger 2024). Diffusion Tensor Imaging (DTI) studies have shown white matter alterations in the middle cerebellar peduncle, corticospinal tract, and cingulum, along with an important role of the right anterior thalamic radiation. Diffusion metrics in these regions, particularly the mean diffusivity of the anterior thalamic radiation, may help predict mild cognitive impairment in MSA patients (Pang et al. 2024). Other studies have found that a reduction in fractional anisotropy in the anterior corpus callosum is related to cognitive impairment in MSA (Hara et al. 2018). Additionally, the sT1w/T2w ratio in white matter constitute an independent predictor for cognitive decline (Sugiyama et al. 2021). In terms of behavioral alterations, widespread fronto-striatal white matter fractional anisotropy reduction has been associated with executive dysfunction in MSA-P.

Functional MRI studies have revealed disruptions in connectivity within the cerebro-cerebellar and cortico-striatal networks, which are linked to cognitive dysfunction in MSA. Specifically, alterations in functional connectivity between the cerebellum and the prefrontal cortex have been linked to deficits in verbal fluency and memory, while disconnection within the cerebellum-limbic/temporal circuit has been related to language and visuospatial function impairment. Furthermore, MSA-P patients exhibit increased connectivity between the dentate nucleus and the posterior cingulate cortex compared to Parkinson’s disease patients and healthy controls, while MSA-C patients show increased functional connectivity between cerebellar, temporo-parietal, and ponto-cerebellar regions compared to healthy controls, suggesting a compensatory mechanism (Piramide et al. 2024). Additionally, some studies found reduced connectivity between the thalamus and some key regions involved in attention, memory, and decision-making, such as the anterior cingulate cortex, superior temporal gyrus, and middle frontal gyrus in MSA patients with freezing of gait. Furthermore, abnormal increases in connectivity between the thalamus and structures related to cognitive regulation, such as the inferior parietal lobule, precuneus, and cerebellum, have also been observed. Thus, thalamic dysfunction may play a role in the cognitive symptoms of MSA (Cheng et al. 2022). Regarding behavioral symptoms in MSA, anxiety has been associated with increased functional connectivity between the amygdala and the parietal, orbitofrontal, precuneus, and medial temporal cortices. Additionally, decreased connectivity between the dorsolateral prefrontal cortex and the dentate nucleus may also be associated with executive control and emotional processing (Yao et al. 2017). From a metabolic point of view, MSA-P patients exhibit a significant metabolic reduction in the frontal cortex and striatum on 18F-FDG PET. Striatal metabolism has been identified as the primary determinant of frontal metabolism in these patients, suggesting striatofrontal deafferentation. Regarding cognitive symptoms, striatofrontal dysfunction is believed to impact executive and attentional functions due to the disruption of cortico-striato-thalamo-cortical circuits (Kim et al. 2017). Additionally, a decrease in metabolism in the right superior frontal gyrus has been observed in MSA patients with cognitive decline, with the regional cerebral metabolic rate of glucose in this structure being a potential biomarker (Chen et al. 2025). Neuromelanin studies have detected that reduced neuromelanin in the rostral part of the locus coeruleus in MSA correlates with greater cognitive dysfunction (Pasquini et al. 2025). Regarding behavioral alterations, reduced metabolism in the dorsolateral prefrontal cortex has been observed in relation to executive dysfunction in MSA-P patients (Jellinger 2024).

Additionally, autonomic dysfunction is thought to play a role in cognitive decline in MSA. Indeed, cognitive impairment is more frequent and progresses faster in MSA patients with orthostatic hypotension, and early-onset orthostatic hypotension has been associated with a higher prevalence of cognitive impairment in these patients. Based on data from other synucleinopathies, the mechanism does not appear to involve common neurodegeneration of anatomical structures, nor has a clear relationship been found between orthostatic hypotension and cerebral white matter lesions (Li et al. 2021b; Li et al. 2025; Ruiz Barrio et al. 2023).

## Conclusions

PSP and MSA are increasingly recognized as disorders in which cognitive and behavioral symptoms constitute integral components of the clinical phenotype rather than secondary or late-stage complications. However, the nature, severity, and temporal profile of these manifestations may differ substantially between the two entities. Across studies, PSP is characterized by an early, prominent, and pervasive disruption of frontal–subcortical networks, resulting in a dysexecutive syndrome frequently accompanied by language, visuospatial, and social cognitive deficits, as well as marked apathy and other behavioral symptoms. In contrast, cognitive impairment in MSA is more heterogeneous, generally milder, and often emerges later in the disease course, with executive dysfunction predominating and core language and social cognition relatively preserved. These differences support the clinical utility of cognitive–behavioral profiling as a complementary tool in the differential diagnosis between PSP and MSA, especially when motor features overlap.

Neuroimaging and neuropathological studies further suggest that these distinct clinical profiles reflect partially divergent network vulnerabilities, with predominant prefrontal and frontal–subcortical involvement in PSP and more variable contributions from frontal, subcortical, cerebellar, and limbic systems in MSA. Importantly, behavioral symptoms such as apathy, impulsivity, and affective disturbances appear closely linked to these network disruptions and may progress independently of motor decline, underscoring their relevance for functional outcome and quality of life.

Taken together, these observations argue for a more systematic incorporation of cognitive and behavioral assessment into the routine evaluation of patients with APS, a process that is increasingly supported by the availability of PSP-specific bedside instruments and structured clinical assessments. Future research should aim to refine disease-specific cognitive markers and develop tailored cognitive and neuropsychiatric assessment tools, particularly for MSA, building on recent advances such as PSP-specific bedside instruments. In addition, longitudinal studies are needed to clarify the evolution of cognitive-behavioral symptoms and their relationship to structural and functional network changes. Such efforts will be essential to improve early diagnosis, prognosis, and patient-centered management strategies in PSP and MSA.
